# Cord blood neutrophils display a galectin-3 responsive phenotype accentuated by vaginal delivery

**DOI:** 10.1186/1471-2431-13-128

**Published:** 2013-08-21

**Authors:** Martina Sundqvist, Veronica Osla, Bo Jacobsson, Anna Rudin, Karin Sävman, Anna Karlsson

**Affiliations:** 1 Department of Rheumatology and Inflammation Research, Sahlgrenska Academy at the University of Gothenburg, Gothenburg, Sweden; 2 Department of Obstetrics and Gynecology, Sahlgrenska University Hospital, Gothenburg, Sweden; 3 Perinatal Centre, Department of Physiology and Neuroscience and Department of Pediatrics, Sahlgrenska Academy at the University of Gothenburg, Gothenburg, Sweden

**Keywords:** Cord blood, Galectin-3, Neonatal immunity, Neutrophils, Priming, Reactive oxygen species

## Abstract

**Background:**

Term neonates are at increased risk of infections due to undeveloped immune mechanisms, and proper neutrophil function is important for perinatal immune defence. Galectin-3, an endogenous β-galactoside-binding lectin, is emerging as an inflammatory mediator and we have previously shown that primed/activated, but not resting, adult neutrophils respond to this lectin by production of reactive oxygen species (ROS). We investigated if galectin-3 is of importance in perinatal immune defence, focusing on plasma levels and neutrophil responsiveness.

**Methods:**

Neutrophils were isolated from peripheral blood of healthy adults and cord blood (CB) after elective Caesarean section (CSCB) and vaginal delivery (VDCB). ROS production was measured by chemiluminescence, L-selectin expression by flow cytometry, and interleukin-8 (IL-8) and galectin-3 concentrations by ELISA. Statistical evaluations were performed using the Mann–Whitney test.

**Results:**

In response to galectin-3, CSCB neutrophils showed a small but clear ROS production not evident in adult cells, signifying that neonatal neutrophils exist in a primed state. IL-8 production was elevated in CSCB cells while L-selectin exposure was equal to adult cells. Comparing CSCB to VDCB neutrophils, the latter showed an extensive galectin-3 responsiveness, indicating that the degree of priming is dependent on mode of delivery. VDCB neutrophils were increasingly prone to shed L-selectin, while the amount of IL-8 was similar to CSCB cells. The endogenous galectin-3 levels were higher in neonatal as compared to adult plasma, unaffected by mode of delivery.

**Conclusions:**

Neutrophils enter a pre-primed state already in the fetus. Upon exposure to the inflammatory stimuli that are associated with labor, the neutrophils develop a reactive phenotype with extensive priming features.

## Background

Term neonates have an increased susceptibility to infection due to incompletely developed defence mechanisms, both in the innate and acquired immunity [1]. Being the first line of defence, the innate immune response and thus the neutrophil granulocyte is of outmost importance for the neonatal protection against infection. Neonatal neutrophils have been demonstrated to behave abnormally as compared to adult cells with regard to basic functions such as transmigration and metabolic activity [2,3].

In healthy adults, neutrophils circulate in a quiescent state in the bloodstream until being recruited to sites of inflammation or infection. During extravasation, the neutrophil phenotype changes into a hyperresponsive, or primed, state that *in vitro* can be induced by a number of agonists, e.g., LPS and G-protein coupled receptor (GPCR) ligands [4,5]. The hyperresponsiveness to stimulation associated with primed neutrophils, is in part due to increased receptors on the cell-surface [6-8].

We have previously shown that term neonatal cord blood neutrophils isolated after vaginal delivery display an increased oxidative response to the chemoattractant fMLF as compared to adult cells [9], an effect not seen after delivery by Caesarean section (CS) [10]. This indicates that neonatal neutrophils in a non-induced, control setting (CS delivery) are similar to healthy adult neutrophils, having the capacity to become primed in response to the inflammatory elicitation that labor and vaginal delivery provides [2,11,12].

A functional difference between resting and primed adult neutrophils is that only primed cells respond to galectin-3, an endogenous β-galactoside binding lectin [4,5,13]. Galectin-3 has emerged as an important marker in several settings of inflammation [14-17]. In the context of perinatal biology, we have found that galectin-3 contributes to brain injury in a murine model of hypoxic ischemia [18] and is found in increased amounts in asphyxiated infants with abnormal outcome [19]. Also, a recent study described a positive correlation of galectin-3 levels with gestational age [20]. The fact that neonatal neutrophils under certain circumstances are primed implies that neonatal cells may be responsive to galectin-3, and that the response may depend on mode of delivery. The aim of our study was thus to investigate if galectin-3 plays a role in the perinatal immune response by interacting with neutrophils.

To provide a basis for our studies, we first investigated the content of galectin-3 in adult and neonatal plasma. Secondly, we established data on how resting neonatal neutrophils (CS delivery) compare to adult neutrophils with regards to priming-related features. And thirdly, we compared the resting neonatal neutrophils to cells from neonates having experienced a vaginal delivery after spontaneous onset of labor. We found that neonatal neutrophils from cord blood after CS showed a small but clear galectin-3 responsiveness while adult neutrophils were inert, indicating a slightly primed phenotype. Cord blood neutrophils isolated after vaginal delivery were substantially more responsive to galectin-3 than their CS counterparts. We thus suggest that neonatal neutrophils in circulation display a preparedness for the challenges that the young host is about to encounter during delivery, and that these cells have the ability to rapidly respond to the inflammatory stimuli accompanying the labor process by taking on a primed phenotype. In fact, endogenous galectin-3 may be such a stimulus contributing to the neonatal immune response, as we found the levels of galectin-3 in cord blood plasma to be constitutively elevated as compared to adult plasma.

## Methods

### Materials

Dextran was purchased from Pharmacosmos (Holbaek, Denmark) and Ficoll-Paque from Fischer Scientific GTF AB (Gothenburg, Sweden). The galectin-3 enzyme-linked immunosorbent assay (ELISA) was from BG Medicine (Waltham, MA, USA) and the human Interleukin-8 (IL-8) DuoSet ELISA was from R&D Systems (Minneapolis, USA). Luminol, isoluminol, and lipopolysaccharide (LPS) from *Escherichia coli* (*E. coli*; serotype 0111:B4), were from Sigma (St Louis, MO, USA). Horseradish peroxidase (HRP), superoxide dismutase (SOD), and catalase were from Boehringer Mannheim (Mannheim, Germany). The phycoerythrin (PE)-conjugated anti-CD62L (L-selectin) monoclonal antibody (mAb) was from Becton Dickinson (San Jose, CA, USA) and the RPMI 1640 and penicillin/streptomycin (PEST) was from PAA Laboratories (Gothenburg, Sweden). Pefabloc and paraformaldehyde (PFA) was from Roche Diagnostics (Indianapolis, USA) and Triton X-100 was from Merck (New Jersey, USA).

### Samples

Heparinized cord blood (CB; 2-15 ml) was obtained from term neonates of gestational age ≥ 37 weeks, at the Sahlgrenska University Hospital (Gothenburg, Sweden). Blood was drawn immediately after the umbilical cord blood was clamped and cut, by needle aspiration of the umbilical vein. Samples were collected from infants delivered by elective Caesarean section (CSCB; n = 18) due to benign causes such as breech presentations, or by vaginal delivery with spontaneous onset of labor (VDCB; n = 20). Separate donors were used for isolation of blood cells (CSCB, n = 10; VDCB; n = 12) and for preparation of plasma (CSCB, n = 8; VDCB; n = 8). Both modes of delivery were performed under regional anaesthesia. All mothers included were previously healthy, with no pregnancy-related complications, and with no signs of infection at delivery. No signs of infection were detected in any of the infants. Ethical approval was obtained from the Regional Ethical Review Board (Gothenburg, Sweden) and informed consent was obtained from parents in accordance with the Declaration of Helsinki. Peripheral blood obtained from healthy adult volunteers (adult; n = 33) was used as control.

For plasma preparation, blood was centrifuged on Ficoll-Paque at 900 × *g* for 20 min, after which the cell-free upper phase was collected, centrifuged and stored at -80°C.

### Measurements of galectin-3 levels in plasma

Galectin-3 levels in plasma were measured using the BGM Galectin-3 ELISA according to manufacturer’s instructions [21].

### Isolation and in vitro priming of human neutrophils

Neutrophils were isolated from heparinized blood as described [22], diluted to 1 × 10^7^ cells/mL in Krebs-Ringer phosphate buffer (KRG, pH 7.3) with Ca^2+^ (1 mM) and stored on ice until use within 4 hours.

Priming of neutrophils was achieved by incubating the cells in the absence or presence of LPS (10 μg/ml) at 37°C for 30 minutes [4].

### Production of recombinant galectin-3

Recombinant human galectin-3 was produced in *E. coli* and purified as described [5,23].

### Production of reactive oxygen species

Neutrophil NADPH-oxidase activity was measured using a luminol/isoluminol-amplified chemiluminescence (CL) system [24]. Briefly, neutrophils (1 × 10^6^) in KRG with Ca^2+^ (1 ml) were equilibrated in a six channel Biolumat LB 9505 instrument (Berthold Co. Wildbad, Germany) for 5 minutes at 37°C, prior to stimulation with galectin-3 (20-40 μg/ml). For detection of extracellular reactive oxygen species (ecROS) the vials contained isoluminol (5 × 10^-5^ M) and HRP (4 U/ml) and for intracellular ROS (icROS) production, vials contained luminol (5 × 10^-5^ M), SOD (50 U/ml) and catalase (2000 U/ml).

### Cell surface expression of L-selectin (CD62L)

After different treatments, neutrophils (1 × 10^7^ cells/ml) were fixated with PFA (2%) for 5 minutes at room temperature, washed twice in PBS and stained with PE-conjugated anti-CD62L mAb (diluted 1/40), for 60 minutes, in darkness, at 4°C. Cells were washed and 10 000 gated neutrophils [FCS (size) vs. SSC (density)] were analysed for L-selectin expression using a FACScan and FlowJo software. Geometric mean fluorescence intensity (MFI) values were used for comparisons.

### Quantification of IL-8 production by isolated neutrophils

Isolated neutrophils (5 × 10^6^ cells/ml) were incubated in RPMI 1640 medium supplemented with 10% heat-inactivated fetal calf serum (10%) and 50 U/mL PEST (1%) at 37°C, 5% CO_2_ for 24 hours, in the absence or presence of LPS (10 μg/ml). The samples were treated with Pefabloc (1 mM), lysed by Triton X-100 (1%), and stored at -80°C until analysis of IL-8 content using a human IL-8 DuoSet ELISA kit, according to manufacturer’s instructions.

### Statistical analysis

Statistical calculations were performed using GraphPad Prism software version 6.0a. A Mann-Whitney test was applied for comparison between groups. A p-value of less than 0.05 was regarded as statistically significant and is indicated by (*) p < 0.05, (**) p < 0.01, (***) p < 0.001 and (****) p < 0.0001. Data are presented as scatter plots with the median for each group indicated by a horizontal line, or as Box and Whisker plots, representing minimum to maximum values, with the box extending from the 25th and 75th percentiles and the median shown as a line in the box.

## Result

### Increased levels of galectin-3 in cord blood plasma after CS as compared to adult plasma

**Figure 1:**
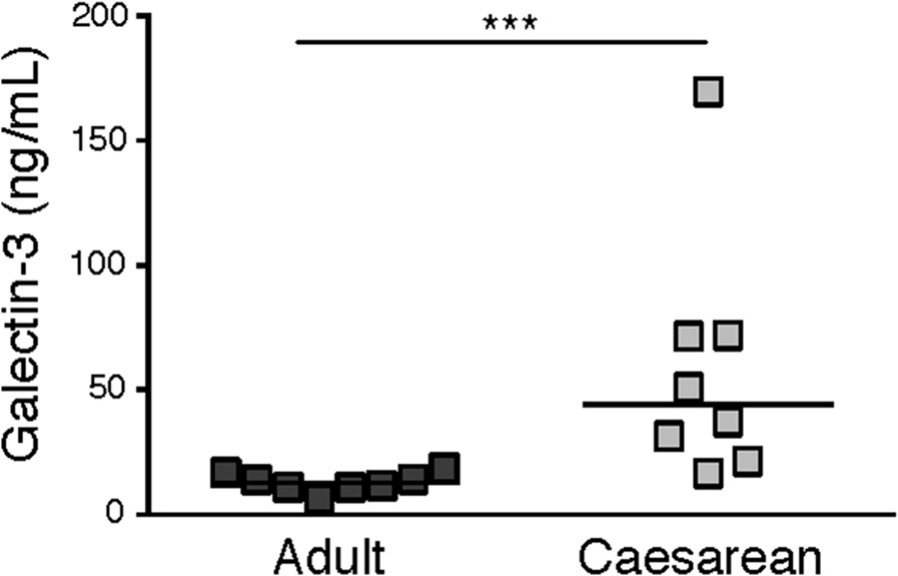
**Galectin-3 levels in plasma.** The concentration of galectin-3 (ng/mL; y-axis) was analysed by ELISA, in plasma from healthy adults (dark grey; n = 8) and from cord blood after elective Caesarean section (light grey; n = 8). Statistical analysis was performed using the Mann–Whitney test (*** p < 0.001).

Galectin-3 levels in healthy adult plasma average at 12 ng/ml [15,21] and increases 3 to 5-fold as a result of different pathologies and inflammatory processes [14-17]. No reports have so far been published on the normal galectin-3 levels in neonatal plasma, why we evaluated the amount in cord blood plasma after CS. We found the levels in neonatal plasma to be significantly elevated as compared to adult plasma (Figure 1).

### Cord blood neutrophils isolated after CS are responsive to galectin-3

Previous studies by us and others show that galectin-3 induces production of ROS in primed but not resting adult peripheral blood neutrophils [4,5,13]. To investigate whether cord blood neutrophils were responsive to galectin-3, i.e., if they were primed already in circulation, and whether the cells were primeable *in vitro*, CSCB neutrophils were examined in comparison to adult cells.

**Figure 2:**
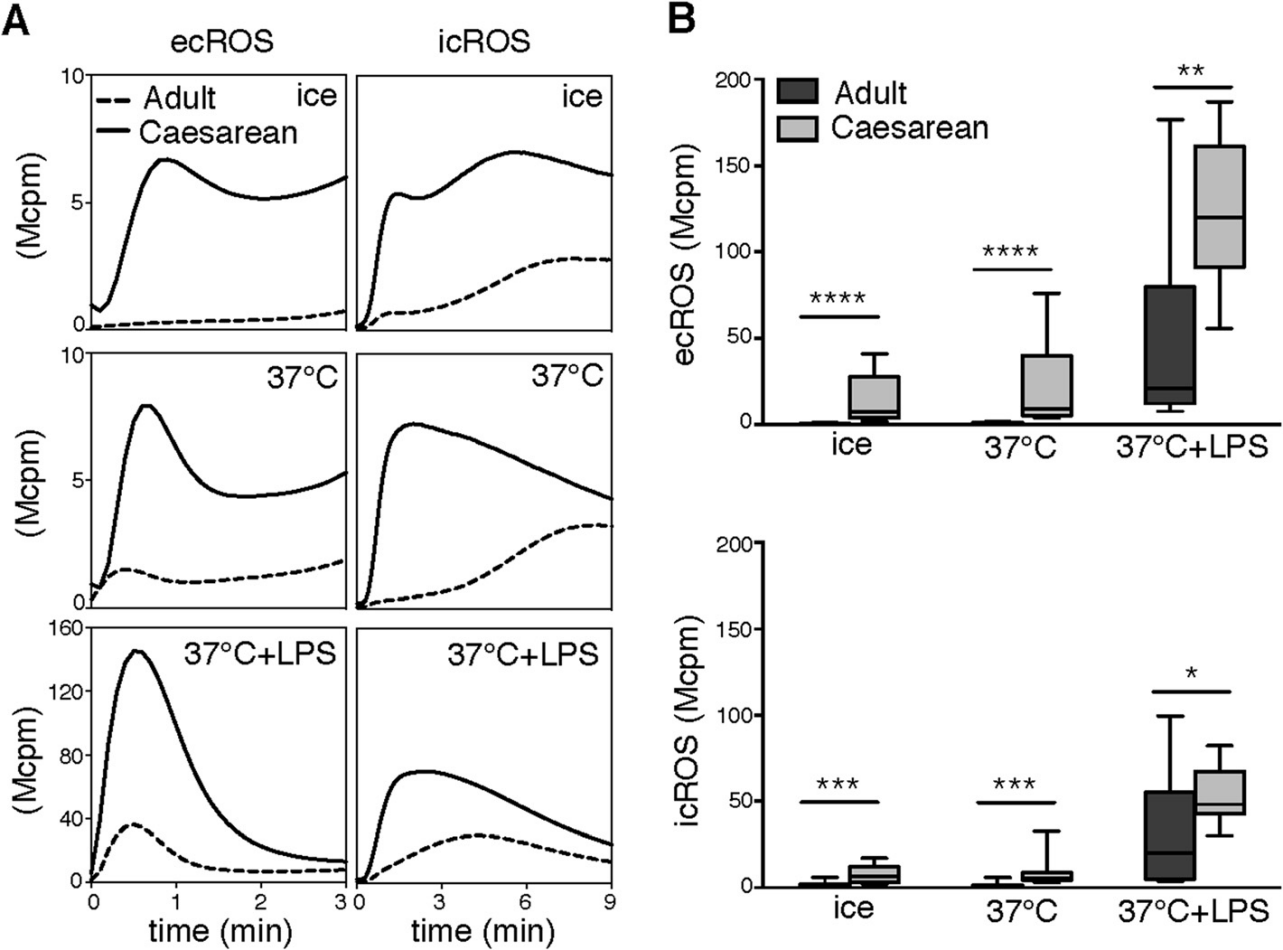
**Galectin-3-induced ROS production.** Isolated neutrophils were kept on ice or incubated at 37°C in the absence or presence of LPS before addition of galectin-3 (at time = 0 min). **(A)** Representative traces of ecROS and icROS production (y-axis, Mcpm; observe the different axes in upper, middle, and lower panels) measured over time (x-axis, minutes) by CL in neutrophils isolated from an adult control (dashed line) and cord blood drawn after a Caesarean section (solid line) are shown. **(B)** Comparisons of galectin-3-induced peak ROS responses between neutrophils isolated from adults (dark grey; n = 10) and cord blood after Caesarean section (light grey; n = 10) following the different incubations are shown for ecROS (upper panel) and icROS (lower panel). Statistical analysis was performed using the Mann–Whitney test (*p < 0.05; **p < 0.005; ***p < 0.001; ****p < 0.0001).

As previously shown, resting adult neutrophils were unresponsive to galectin-3, while their LPS-primed phenotype responded readily [5] (Figure 2A). Interestingly, for the CSCB neutrophils the picture was different. The non-stimulated cells responded (Figure 2A) at levels slightly above but significantly increased as compared to adult neutrophils (Figure 2B).

After mild priming (37°C) the trend was the same, and after LPS-priming the CSCB neutrophils produced vast amounts of galectin-3-induced ROS as compared to adult neutrophils (Figure 2B). These data indicate that CSCB neutrophils are in a state of pre-priming in circulation, making the cells slightly responsive to galectin-3 and by far more easily primed than adult cells.

**Figure 3:**
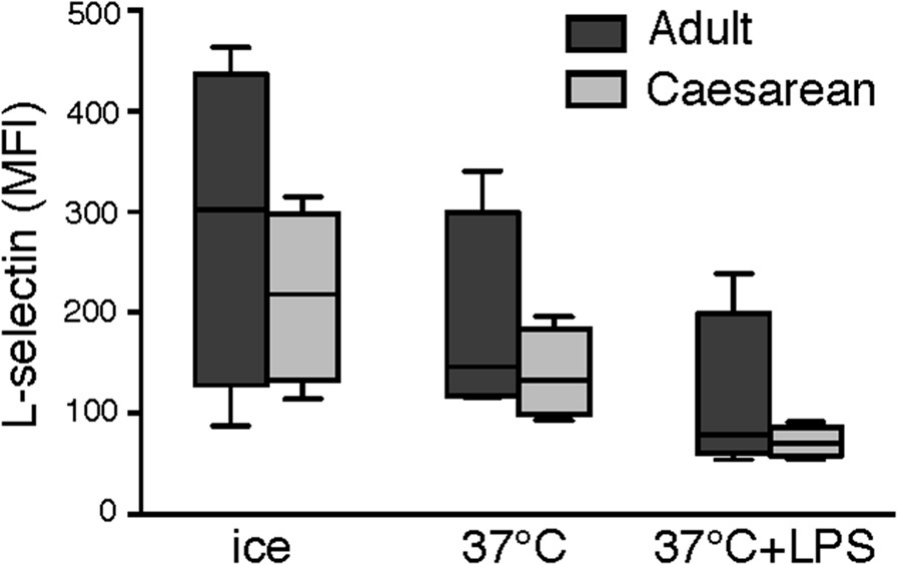
**Cell-surface expression of L-selectin.** Neutrophils isolated from adults (dark grey; n = 4) and cord blood after Caesarean section (light grey; n = 4) were kept on ice or incubated at 37°C in the absence or presence of LPS (10 μg/ml) and investigated for cell-surface expression of L-selectin by flow cytometry. The geometric mean fluorescence intensities (y-axis, MFI) are shown and statistical analysis was performed using the Mann–Whitney test. No statistically significant differences were found between adult and neonatal neutrophils at any of the conditions.

In adult neutrophils, priming of the galectin-3-induced ROS response correlates with shedding of L-selectin [5,6,8]. However, CSCB neutrophils expressed equal levels of L-selectin as adult cells (in resting state) and were as prone to shed their L-selectin in response to *in vitro* priming as adult cells, both after mild priming (37°C) and after priming with LPS (Figure 3).

### Increased IL-8 production in CSCB neutrophils as compared to adult neutrophils

**Figure 4:**
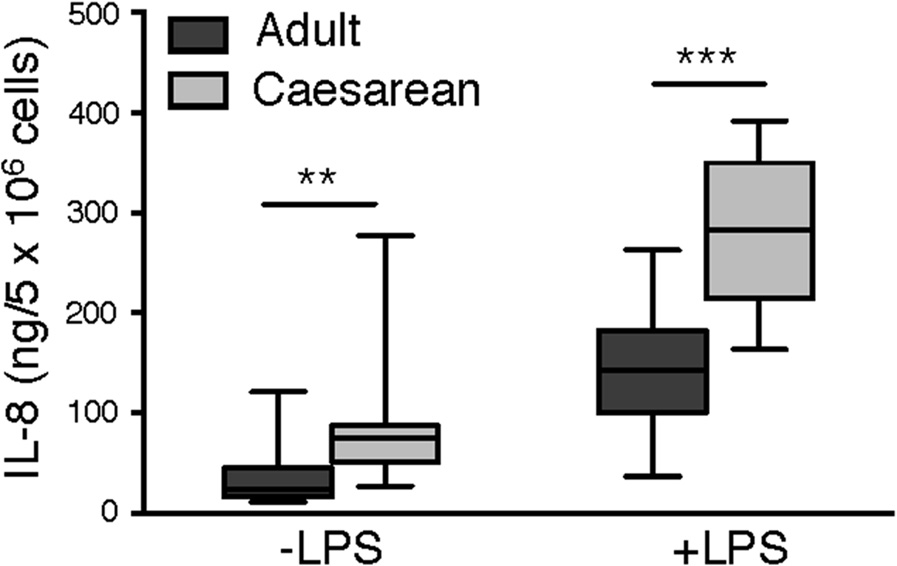
**IL-8 production by neutrophils.** Neutrophils isolated from adults (dark grey; n = 10) and from cord blood after Caesarean section (light grey; n = 10) were incubated for 24 hours at 37°C in the absence or presence of LPS (10 μg/ml). The concentration of IL-8 was analysed in the lysates by ELISA. Statistical analysis was performed using the Mann–Whitney test (**p < 0.005; ***p < 0.001).

The production of IL-8, a potent neutrophil chemoattractant produced by LPS-treated neutrophils [6,25], was investigated in CSCB and adult neutrophils. Even in the absence of LPS treatment, the CSCB neutrophils produced significantly more IL-8 compared to adult cells (Figure 4). LPS treatment, increased IL-8 production in both populations, with CSCB cells again showing significantly higher levels (Figure 4).

These data support that the phenotype of cord blood neutrophils from a non-induced, control setting (CS delivery) differs significantly from that of adult cells, being in a pre-primed state and more responsive to pro-inflammatory stimulus already in the fetus, prior to experiencing the inflammatory stimuli associated with labor.

### Vaginal delivery renders cord blood neutrophils primed in circulation

**Figure 5:**
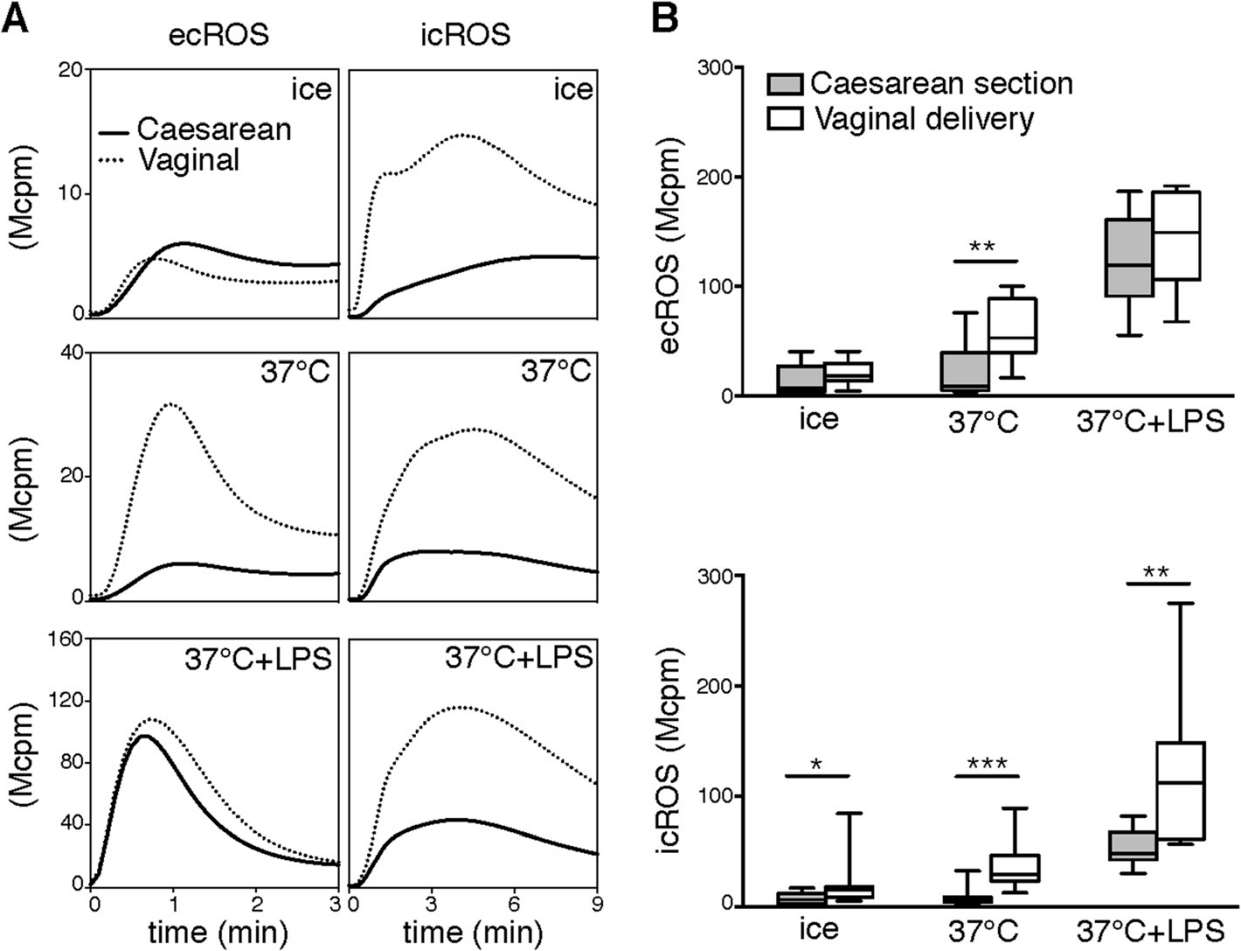
**Galectin-3-induced ROS-production in neutrophils from cord blood obtained after Caesarean section or vaginal delivery.** Isolated cord blood neutrophils were kept on ice or incubated at 37°C in the absence or presence of LPS before addition of galectin-3 (at time = 0 min). **(A)** Representative traces of ecROS and icROS production (y-axis, Mcpm; observe the different axes in upper, middle, and lower panels), measured over time (x-axis, minutes) by CL, are shown for neutrophils from Caesarean section (black lines) and vaginal delivery (dotted lines). **(B)** Comparisons of galectin-3-induced peak ecROS (upper panel) and icROS (lower panel) responses between cord blood neutrophils from Caesarean section (light grey; n = 10) and vaginal delivery (white; n = 10) after the different incubations are shown. Statistical analysis was performed using the Mann–Whitney test (*p < 0.05; **p < 0.005; ***p < 0.001).

Having determined that CSCB neutrophils differ phenotypically from adult cells, we investigated how the inflammatory setting associated with labor and vaginal delivery affected the neutrophil phenotype, comparing CSCB to VDCB neutrophils. With regard to galectin-3-induced ecROS production, CSCB and VDCB neutrophils responded equally well with the exception for cells exposed to mild priming (37°C), where VDCB neutrophils produced significantly more ecROS (Figure 5A, B). However, for the galectin-3-induced icROS production, the response was significantly increased in VDCB neutrophils compared to CSCB cells, both without and after *in vitro* priming (Figure 5A, B). Hence, the VDCB neutrophils were to a higher extent primed and thus potentially more inflammatory in their phenotype as compared to CSCB cells.

This was in line with that the VDCB neutrophils displayed extensive cleavage of L-selectin already after incubation at 37°C as compared to the CSCB cells, an effect that was even more pronounced after LPS treatment (Table 1). Thus, the primed response to galectin-3 by VDCB neutrophils is accompanied by cleavage of L-selectin, in contrast to CSCB cells which, although showing a pre-primed galectin-3 response, exhibited a resting phenotype with regard to L-selectin exposure, equal to circulating adult neutrophils.

**Table 1:** Comparison of neutrophil activation features and inflammatory markers between CSCB and VDCB

**Laboratory parameters**	^**a**^**CSCB**	^**b**^***p*-value**	**VDCB**	**Sample size (CSCB/VDCB)**
**Neutrophil L-selectin expression (MFI)**				
ice	^c^218 (114–315)	ns	116 (91–213)	4/6
37°C	133 (93–196)	**	62 (51–77)	4/6
37°C + LPS	71 (54–91)	**	43 (40–49)	4/6
**IL-8 production (ng/5 × 10**^**6 **^**neutrophils)**				
- LPS	75 (27–277)	ns	51 (8–292)	10/11
+ LPS	282 (164–392)	ns	254 (138–619)	10/11
**Galectin-3 (ng/ml)**				
plasma	44 (17–170)	ns	31 (21–39)	8/8

^a^abbreviations: *CSCB*, Caesarean section cord blood; *VDCB*, vaginal delivery cord blood.

^b^Statistical analysis was performed by the Mann–Whitney test (ns = no statistically significant difference; **p < 0.005).

^c^Data is presented as median (range).

Both neutrophil production of IL-8 and plasma levels of galectin-3 were similar between VDCB and CSCB (Table 1), indicating that these inflammatory mediators are constitutively increased in neonates independent on mode of delivery.

## Discussion

This study was undertaken to investigate neutrophil priming status and galectin-3 involvement in perinatal blood circulation. We have previously shown that adult neutrophils interact with galectin-3 only after extravasation into an inflamed tissue [5]. The neutrophil-galectin-3 interaction is regulated by transferring specific galectin-3 receptors from intracellular granules to the cell surface upon pre-activation/priming, of the neutrophil, rendering the cell fully responsive, while the unprimed cell is inert [4,5,13]. Neutrophil priming *in vivo* is most often associated with the transmigration process [6,7], however, primed neutrophils can be found in circulation, as shown for e.g., systemic inflammatory response syndrome [26].

A main finding in this study is that resting neonatal neutrophils display a phenotype different from adult neutrophils. The CSCB neutrophils were more responsive to galectin-3 in the absence of *in vitro* priming. After priming with LPS, both CSCB and adult neutrophils responded to galectin-3, although CSCB cells again responded significantly stronger, indicating that the neonatal phenotype includes being pre-primed to galectin-3 stimulation, possibly associated with granule mobilization, which is a prerequisite for galectin-3-responsiveness in adult neutrophils [5]. This pre-primed state was not accompanied by L-selectin shedding, a priming feature not directly related to degranulation but to activation of surface proteases [27], and we therefore conclude that galectin-3 responsiveness (possibly associated with degranulation) is a more sensitive priming determinant than L-selectin shedding.

We also compared how labor and vaginal delivery affects the neonatal neutrophil responsiveness to galectin-3 and found the VDCB neutrophils much more responsive than the CSCB cells. In fact, VDCB neutrophils were already more primed after isolation from whole blood, and were further primed by mere incubation at 37°C. After incubation with LPS, CSCB and VDCB neutrophils were equally effective in releasing galectin-3-induced ecROS, however, galectin-3-induced icROS production was much more enhanced in the VDCB neutrophils as compared to CSCB cells. Production of icROS (in the absence of phagosome formation) is of importance for the regulation of inflammation [24], and an imbalanced production of icROS can be correlated to different inflammatory conditions, e.g., the autoinflammatory disorder Synovitis Acne Pustulosis Hyperostosis and Osteitis (SAPHO, decreased icROS production) [28], a novel form of Chronic Granulomatous Disease (CGD, decreased or absent icROS production) [29] and the periodic fever syndrome Periodic Fever Aphthous stomatits Pharyngitis and cervical Adenitis (PFAPA, increased icROS production) [30]. The fact that the VDCB neutrophils showed an enhanced galectin-3-induced icROS production as compared to the CSCB neutrophils is in agreement with the primed phenotype and thus the pro-inflammatory state of these neutrophils as described by us and others [2,9,10,12].

We show increased production of IL-8 in CSCB neutrophils as compared to adult cells, in line with previous studies [31]. The IL-8 production was equal between CSCB and VDCB neutrophils, indicating that neonatal neutrophils produce IL-8 at a constitutively enhanced rate, giving support to the notion that they are in a pre-inflammatory state already in circulation, prepared for the challenges that encounter with the outer world, including pathogens, provides.

We found the galectin-3 concentration in cord blood plasma to be significantly increased as compared to adults, and the levels were equally high in CSCB and VDCB plasma, indicating that the galectin-3 levels in plasma are not altered by the same mechanisms that drive neutrophil priming, but is a delivery-independent feature in the circulation of the fetus at a time point close to delivery. Such increases in circulating galectin-3 have been described in several pathological and inflammatory processes, but never before in neonates [14-17]. The role of galectin-3 in neonatal plasma can only be speculated on. It may be part of the preparation (pre-priming) of the neonate and its cells and tissues for an inflammatory challenge, i.e., delivery. A recent study show positive correlation between galectin-3 levels and gestational age, as well as increased galectin-3 levels after incubating cord blood cells with group B streptococci, suggesting that galectin-3 plays a role in the neonatal innate immune defence against microbes [20]. We and others have shown that galectin-3 functions as an opsonin by promoting clearance of bacteria [32], red blood cells [33], and apoptotic neutrophils [34]. These features resemble those of an activated complement system, i.e., providing opsonisation and immunomodulation. Infants show reduced levels of complement components and complement activation [3] and increased levels of galectin-3 could very well play a compensatory role.

## Conclusions

Cord blood neutrophils from a non-induced, control setting (neonates delivered by CS) display a phenotype different from their adult counterpart, being slightly primed as measured by responsiveness to galectin-3. After labor and vaginal delivery the neonatal neutrophils display a clearly primed phenotype, seen as pronounced responsiveness to galectin-3 and extensive cleavage of L-selectin. The differences in phenotype between circulating neutrophils in (CS) neonates as compared to adults brings new information on the neonatal innate immune defence. Speculatively, the altered neutrophil phenotype in newborns appearing already before labor may be a preparation for the encounter with microbial (maternal commensal flora) and inflammatory challenges that occur during delivery, and this primed state is then further accentuated during vaginal delivery corresponding to the associated increased risk of infection. The differences in neutrophil physiology after delivery by CS and VD may in fact be of importance during the neonate’s first interaction with the outer world; possibly, neonates delivered vaginally may have an increased initial preparedness for this encounter. Newborns also demonstrated increased levels of circulating galectin-3, irrespective of delivery mode. The importance of galectin-3 in tuning the immune response during delivery warrants further investigation.

## Abbreviations

CS: Caesarean section; CSCB: Caesarean section cord blood; CL: Chemiluminescence; E. coli: Escherichia coli; ELISA: Enzyme-linked immunosorbent assay; fMLF: N-formylmethionyl-leucyl-phenylalanine; HRP: Horseradish peroxidase; IL-8: Interleukin 8; KRG: Krebs-Ringer phosphate buffer; LPS: Lipopolysaccharide; NADPH-oxidase: Nicotinamide adenine dinucleotide phosphate-oxidase; Mcpm: Mega (10^6^) counts per minute; MFI: Geometric mean fluorescence; PBS: Phosphate buffered saline; PE: Phycoerythrin; PEST: Penicillin/streptomycin; PFA: Paraformaldehyde; ROS: Reactive oxygen species; SOD: Superoxide dismutase; VDCB: Vaginal delivery cord blood.

## Competing interest

The authors declare that they have no competing interests.

## Authors’ contribution

AK, KS and BJ designed the research. KS, BJ and AR recruited cord blood samples. VO and MS performed the experiments. MS conducted the statistical analysis. AK, VO, KS and MS interpreted the data, and MS, AK, BJ, AR and KS wrote the manuscript. All authors read and approved the final manuscript.

## Pre-publication history

The pre-publication history for this paper can be accessed here:

http://www.biomedcentral.com/1471-2431/13/128/prepub
